# Food composition tables in resource-poor settings: exploring current limitations and opportunities, with a focus on animal-source foods in sub-Saharan Africa

**DOI:** 10.1017/S0007114516003706

**Published:** 2016-11-08

**Authors:** Julia de Bruyn, Elaine Ferguson, Margaret Allman-Farinelli, Ian Darnton-Hill, Wende Maulaga, John Msuya, Robyn Alders

**Affiliations:** 1 Faculty of Veterinary Science, University of Sydney, Sydney, NSW 2006, Australia; 2 Charles Perkins Centre, University of Sydney, Sydney, NSW 2006, Australia; 3 School of Life and Environmental Sciences, University of Sydney, Sydney, NSW 2006, Australia; 4 London School of Hygiene and Tropical Medicine, London WC1E 7HT, UK; 5 The Boden Institute of Obesity, Nutrition, Exercise & Eating Disorders, University of Sydney, Sydney, NSW 2006, Australia; 6 Tanzania Veterinary Laboratory Agency, Dar es Salaam, Tanzania; 7 Sokoine University of Agriculture, Morogoro, Tanzania; 8 International Rural Poultry Centre, Kyeema Foundation, Brisbane, QLD 4000, Australia

**Keywords:** Food security, Under-nutrition, Animal-source foods, Food composition tables, Nutrition-sensitive interventions

## Abstract

Animal-source foods (ASF) have the potential to enhance the nutritional adequacy of cereal-based diets in low- and middle-income countries, through the provision of high-quality protein and bioavailable micronutrients. The development of guidelines for including ASF in local diets requires an understanding of the nutrient content of available resources. This article reviews food composition tables (FCT) used in sub-Saharan Africa, examining the spectrum of ASF reported and exploring data sources for each reference. Compositional data are shown to be derived from a small number of existing data sets from analyses conducted largely in high-income nations, often many decades previously. There are limitations in using such values, which represent the products of intensively raised animals of commercial breeds, as a reference in resource-poor settings where indigenous breed livestock are commonly reared in low-input production systems, on mineral-deficient soils and not receiving nutritionally balanced feed. The FCT examined also revealed a lack of data on the full spectrum of ASF, including offal and wild foods, which correspond to local food preferences and represent valuable dietary resources in food-deficient settings. Using poultry products as an example, comparisons are made between compositional data from three high-income nations, and potential implications of differences in the published values for micronutrients of public health significance, including Fe, folate and vitamin A, are discussed. It is important that those working on nutritional interventions and on developing dietary recommendations for resource-poor settings understand the limitations of current food composition data and that opportunities to improve existing resources are more actively explored and supported.

Under-nutrition remains a pervasive issue of the current century, with profound implications for individual growth, development and survival, incidence of acute and chronic diseases, and national economic productivity and wealth^(^ ^1^ ^)^. The latest global estimates suggest 795 million people to be chronically undernourished, with substantial geographic variation in progress towards international development goals^(^ ^2^ ^)^. The spectrum of nutrition-related disorders includes wasting, stunting and micronutrient deficiencies, with the prevalence of stunting being a critical indicator of progress in child survival, reflecting long-term exposure to suboptimal health and nutrition^(^ ^3^ ^)^. An increasing incidence of overweight, obesity and non-communicable diseases is also emerging in many low- and middle-income countries (LMIC), where the coexistence of under- and over-nutrition now occurs and is termed the ‘double burden of malnutrition’^(^ ^4^ ^)^.

There is an increasing focus on the role of nutrition-sensitive interventions, including food-based approaches, which use locally available and culturally acceptable products, to achieve sustainable improvements in human nutrition. Animal-source foods (ASF) are known to provide protein of high biological value and micronutrients such as Fe, Zn and vitamin B_12_ that are difficult or impossible to obtain in adequate amounts from plant-source foods alone^(^ ^5^ ^,^ ^6^ ^)^. Despite concern by some about the environmental and nutritional implications of increased ASF consumption predicted to accompany population and income growth, there is scope for ASF to enhance the nutrient adequacy of traditional diets based on cereals and tubers in resource-poor settings^(^ ^5^ ^–^ ^8^ ^)^. The inclusion of ASF in the diet has been shown to promote growth and improve cognitive function, physical activity and health^(^ ^9^ ^–^ ^11^ ^)^, and among pregnant women it has been shown to support fetal growth and development and enhance maternal nutrition in preparation for lactation^(^ ^12^ ^)^.

The development of meaningful food-based recommendations for dietary improvement relies on an assessment of existing diets, an understanding of the food resources accessible to a given population, the population’s nutrient requirements and accurate data on the nutrient content of available food items^(^ ^13^ ^)^. Measurement error or incorrect assumptions occur at all stages of dietary assessment, including misreporting habitual food intakes, elevated nutrient requirements due to high levels of infection and errors in food composition data. In this article, we focus on systematic errors present in food composition databases that may result in incorrect estimations of dietary adequacy, especially in LMIC populations where the intakes of ASF are increasing^(^ ^14^ ^)^. The merits and limitations of various food security and nutrition indicators and their implications for understanding the nutritional status of individuals and communities^(^ ^15^ ^)^ should be recognised, but are beyond the scope of this article.

Information on the macronutrient and micronutrient content of food items is used both to estimate the nutrient content of current diets and to formulate guidelines for increasing dietary adequacy. Food composition data are available through national and regional food composition tables (FCT) and, increasingly, in electronic formats. Although such resources have value in understanding the effects of diets on health, growth and development and devising diets for individuals and populations, their limitations need to be understood^(^ ^16^ ^)^. In a comparison of three different commonly used data sources of the macronutrient content of foods consumed in Uganda, Baingana^(^ ^17^ ^)^ draws attention to the marked variation in results obtained, and contends that inappropriate food composition data have the potential to ‘undermine or misdirect research or nutrition efforts’.

The International Network on Food Data Systems (INFOODS), led by the FAO, has been tasked with coordinating efforts to improve the quality of food composition data globally. Many LMIC currently lack capacity for nutrient analysis, which limits the availability of country-specific food composition data. It is among the aims of INFOODS to ensure that the large body of available food composition data is of sufficient quality to be combined with directly analysed values. To achieve this, the FAO^(^ ^16^ ^)^ proposes ten criteria for a comprehensive food composition database. The data should be (1) representative, (2) of sound analytical quality, including comprehensive coverage of both (3) foods and (4) nutrients, with (5) clear food descriptions and (6) data should be presented in a consistent, unambiguous manner, (7) showing origins of data at a nutrient level, (8) in an easy-to-use format, which is (9) compatible with existing international standards and (10) has few missing data.

As biological materials, foods exhibit variations in composition. Differences in the nutrient content of a given item may be due to environmental conditions, crop variety or animal breed, stage of maturity, processing methods and cooking techniques^(^ ^16^ ^,^ ^18^ ^)^. In the case of ASF, the composition of products derived from intensively raised animals of commercial breeds may differ from those of indigenous livestock in resource-poor settings, which are typically reared in low-input production systems, sometimes on mineral-deficient soils and commonly not receiving balanced feed. A crucial shortfall of many national or regional food composition databases, including those released in recent years, is their reliance on a small number of existing data sets – often from more high-income nations and sometimes from analyses conducted decades previously.

This article reviews selected FCT currently in use within sub-Saharan Africa, first examining the spectrum of ASF reported and then considering the source of data for each item. Of criteria for reliable composition data proposed by the FAO^(^ ^15^ ^)^, this study focuses primarily on whether databases are representative of national or regional diets and whether data may be considered of sound quality for the context in which they are intended to be used. Using poultry products as an example, comparisons are made between compositional data from three high-income nations, and the implications of using data from different sources are discussed. Focus is given to three micronutrients because their content in ASF items differed markedly between references: vitamin A, which is relevant because of the widespread occurrence of deficiency globally; vitamin B_12_, found naturally only in ASF; and folate, which is of particular relevance to women of reproductive age because of its role in preventing neural tube defects during embryonic development in early pregnancy.

## Methods

The FAO INFOODS directory provides details of available food composition data according to geographic location, as well as international databases. Many of the resources listed are no longer in print and are difficult to access. For the purposes of this analysis, national and regional food composition data for sub-Saharan Africa accessible online in the English language were examined. Databases meeting criteria for inclusion (in reverse chronological order of publication date, from 2012 to 1989) were as follows: *West African Food Composition Table* ^(^ ^19^ ^)^, *A Food Composition Table for Central and Eastern Uganda* ^(^ ^20^ ^)^, *Food Composition Table for Use in The Gambia* ^(^ ^21^ ^)^, *Food Composition Tables for Mozambique* ^(^ ^18^ ^)^, *Tanzania Food Composition Tables* ^(^ ^22^ ^)^, *Lesotho Food Composition Table* ^(^ ^23^ ^)^ and ‘Nutritive value of foods of Zimbabwe’^(^ ^24^ ^)^. Data from the *Food Composition Table for Use in Africa* ^(^ ^25^ ^)^ were also reviewed, but owing to large sections of missing nutrient information they were deemed insufficiently complete for inclusion in the comparative analysis.

Lists of ASF were compiled from each of the selected databases. Meat and meat products, fish and shellfish, milk, eggs and insects were included in both raw and, where available, cooked or processed forms. FCT entries based on recipes that include ASF as an ingredient (e.g. ‘fish relish with coconut milk’) and commercially processed meat-based entries (e.g. sausages, canned tuna) were excluded from this analysis.

ASF items from each reference were divided into seven categories: *meat flesh*, defined as skeletal muscle with any attached fat, connective tissue, nerves, vessels, blood and skin^(^ ^26^ ^)^; *offal* including blood, brain, heart, intestines, kidneys, pancreas, spleen, thymus, tongue and tripe, but excluding meat flesh, bone and bone marrow^(^ ^26^ ^)^; *other carcass components*, covering entries that include bone, such as chicken heads and feet; *fish and shellfish*; *milk*; *eggs*; and *insects*.

National food balance data corresponding to the year of publication of each database were collated for evaluating how effectively the range of ASF items matched the reported food supply^(^ ^27^ ^)^. In the absence of comprehensive national food consumption survey data, food balance sheets are recognised to provide guidance on the domestic availability of foods and their contribution to diets during FCT compilation^(^ ^16^ ^)^. Although the supply of an ASF category will not always be expected to correspond to the number of FCT entries (for example, milk might be widely consumed but represented in a small number of entries), comparisons have been made between the availability of food and its inclusion in FCT lists.

In a second aspect of the analysis, ASF entries were classified according to the data source for each food item, on the basis of types of compositional data as defined by Greenfield & Southgate^(^ ^16^ ^)^. These include *original analytical values*, derived from the published literature or unpublished laboratory reports based on valid methodology and local food items; *imputed values*, whereby data are estimated from analytical values for a similar food or for another form of the same food; *calculated values*, which apply accepted yield factors and nutrient retention factors for the relevant cooking methods to data from other sources; *borrowed data*, drawn from other tables or databases where reference to original sources may or may not be provided; and items of *unspecified* source.

Entries have been further categorised according to the region from which compositional data originated: Africa, the USA, the UK, Europe or Asia. For data borrowed from other FCT, the original sources have been consulted where possible, and it is indicated where this has not been possible. Many of the ASF items listed in the Gambian and West African databases have been compiled from multiple sources, and the degree to which each source has contributed to an entry is often not evident. In order to summarise the origins of data in these cases, all countries contributing to a database entry have been given equal weighting (e.g. 0·5 each if two data sources, 0·33 each if three data sources), and the total number of items from each region has been rounded to whole numbers.

On the basis of a predominance of data from UK and US sources, a third exercise was undertaken comparing nutrient content of selected poultry products in the most recent food composition databases from these two countries with a third data set, from Australia. Data were drawn from the UK’s recently updated *McCance and Widdowson’s: The Composition of Foods Integrated Dataset* ^(^ ^28^ ^)^, the 28th release of the United States Department of Agriculture National Nutrient Database^(^ ^29^ ^)^ and the Australian Food, Supplement and Nutrient Database^(^ ^30^ ^)^. The three sets of published values for macronutrients and selected micronutrients (vitamin A, vitamin B_12_, folate, vitamin E, Fe, Zn and Se) were compared per 100 g edible portion of light and dark chicken meat, chicken liver and whole chicken eggs, in their raw forms.

## Results

### Spectrum of animal-source foods

The databases examined varied substantially in the number of food items included. The comparatively more extensive FCT for West Africa and Central and Eastern Uganda contain 113 and 126 ASF entries, respectively, whereas the Gambian reference includes only thirteen. The Gambian database has a strong emphasis on local recipes and prepared dishes, and reports only a very limited number of individual food items. There is also a notable lack of detail in the description of food items in this reference, such as ‘egg’ and ‘meat, boiled’, both of unspecified livestock species.

Differences can be seen in the number and relative proportion of entries in the seven ASF categories in each of the databases, as outlined in Table 1a. Fish and shellfish constitute a substantially higher proportion of ASF in databases for The Gambia (69·2 %, *n* 9) and Mozambique (55·6 %, *n* 20), compared with Zimbabwe (5·6 %, *n* 3) and Lesotho (*n* 0). In Lesotho, the number of meat flesh entries is matched by the number of offal entries (both 35·3 %, *n* 18), whereas in all other databases there are two or more times more meat flesh than offal entries. Other carcass components, which include chicken heads and feet of chicken, cattle and small ruminants, are only found in the tables for Uganda (5·6 %, *n* 7) and Lesotho (7·8 %, *n* 4).

**Table 1:** Animal-source food (ASF) entries in selected African food composition tables, by category, mode of preparation, animal type, nature of data and origin of data (Number and percentage of total ASF entries)

	Uganda		West Africa		The Gambia		Mozambique		Tanzania		Lesotho		Zimbabwe	
	*n*	%	*n*	%	*n*	%	*n*	%	*n*	%	*n*	%	*n*	%
*(a) ASF categories*														
Meat flesh	49	38·9	42	37·2	2	15·4	10	27·8	10	30·3	18	35·3	30	55·6
Offal	23	18·3	14	12·4	0		1	2·8	5	15·2	18	35·3	10	18·5
Other carcass components	7	5·6	0		0		0		0		4	7·8	0	
Fish and shellfish	31	24·6	45	39·8	9	69·2	20	55·6	11	33·3	0		3	5·6
Milk	3	2·4	5	4·4	1	7·7	3	8·3	1	3·0	4	7·8	4	7·4
Eggs	7	5·6	3	2·7	1	7·7	2	5·6	4	12·1	7	13·7	3	5·6
Insects	6	4·8	4	3·5	0		0		2	6·1	0		4	7·4
*(b) Preparation*														
Raw	37	29·4	43	38·1	6	46·2	24	66·7	17	51·5	27	52·9	39	72·2
Cooked, dried or processed	89	70·6	70	61·9	7	53·8	12	33·3	16	48·5	24	47·1	15	27·8
*(c) Animal type*														
Domestic livestock	89	70·6	61	54·0	4	30·8	15	41·7	20	60·6	51	100	46	85·2
Fish, shellfish	31	24·6	45	39·8	9	69·2	20	55·6	11	33·3	0		3	5·6
Wild foods	6	4·8	7	6·2	0		1	2·8	2	6·1	0		5	9·3
*(d) Nature of data*														
Original analysis	0		0		0		4	11·1	0		0		0	
Imputed	39	31·0	0		2	15·4*	0		0		0		0	
Calculated	0		65	57·5*	0		8	22·2	0		5	9·8	0	
Borrowed	87	69·0	47	41·6*	11	84·6*	24	66·7	0		45	88·2	0	
Not specified	0		1	0·9	0		0		33	100	1	2·0	54	100
*(e) Origin of data*														
Africa	6	4·8	24	21·2†	0		15	41·7‡	0		23	45·1§	0	
USA	112	88·9	35	31·1	0		15	41·7	0		18	35·3	0	
UK	0		9	8·1	4	30·8	1	2·8	0		4	7·8	0	
Europe	0		28	24·5	0		0		0		0		0	
Asia	8	6·3	10	8·8	0		0		0		0		0	
Unknown	0		7	6·2	9	69·2||	5	13·9	33	100	6	11·8	54	100
Total ASF entries	126		113		13		36		33		51		54	

* Multiple data sources for each item.

†‡§ Data from South African databases; original source unable to be traced († 8/24, ‡ 7/15 and § 23/23).

|| 7/9 Entries include data on Ca, P and Zn contents from The Gambia.

Table 2 presents national food balance data for ASF from the year of compilation of each database^(^ ^27^ ^)^. High reported levels of offal supply in Lesotho and Uganda (2·06 and 1·51 kg/capita per year, respectively) are matched by high numbers of offal FCT entries (*n* 18 and *n* 23, respectively), whereas the low reported offal supply in Mozambique and The Gambia (0·58 kg/capita per year for both) is also reflected in their national FCT databases (*n* 1 and *n* 0, respectively). Similar patterns are seen for fish and shellfish, where both the high per capita supply in West Africa and Uganda and the low supply in Lesotho and Zimbabwe are reflected in the number of related FCT entries.

**Table 2:** National food balance data for animal-source foods (ASF) in selected African countries, corresponding to the year of food composition table publication^(^ ^34^ ^)^

	National food balance (kg/capita per year)					
ASF category	Uganda*	The Gambia	Mozambique	Tanzania	Lesotho	Zimbabwe
Meat flesh	12·55	8·65	8·23	8·84	23·2	11·87
Offal	1·51	0·58	0·58	1·14	2·06	1·31
Fish and shellfish	12·95	26·51	8·47	5·09	0·52	2·4
Milk	31·34	59·44	3·12	20·91	16·01	30·37
Eggs	0·97	0·58	1·28	0·67	0·52	1·22

* Food balance data not available for 2012; data for 2011 was used instead.

The range of livestock species whose milk is included in the databases varies, from cattle only (Tanzania and The Gambia) to cattle and goats (Lesotho, Mozambique, Uganda and Zimbabwe) and cattle, goats and camels (West Africa). The majority of FCT provide data only for chicken eggs, with only two references including duck eggs (Lesotho and Mozambique) and one including turkey eggs (Lesotho). Insects including termites, locusts, crickets and caterpillars are found in four of the seven databases examined (Tanzania, Uganda, West Africa and Zimbabwe).

The preparation state of ASF entries is listed as the percentage of food items that were raw, cooked or processed in each FCT (Table 1b). The Zimbabwe database, the oldest reference consulted, includes a majority of food items in their raw form (72·2 %, *n* 39), whereas the newer references report a larger number of items in cooked or processed forms, most prominently in the Ugandan FCT (70·6 %, *n* 89). Databases were also examined for the breadth of animal species contributing to the ASF listed, as presented in Table 1c. Of particular interest was the inclusion of products from non-domesticated animals. No such items are found in resources from The Gambia and Lesotho, but other references, in addition to the aforementioned invertebrates, include entries for rodent (Mozambique), antelope (Mozambique), crocodile (West Africa) and an unspecified ‘game animal’ (West Africa).

### Sources of data

The databases varied in their inclusion of details on the origins of published data, including sampling methodology, analytical techniques and the source of borrowed nutrient values; five of the seven tables (those of Lesotho, Mozambique, The Gambia, Uganda and West Africa) provided an indication of the data source for individual food entries, whereas the remaining two (for Tanzania and Zimbabwe) provided only general information about the data compilation process and a list of references used.

In the Ugandan database, 69·0 % (*n* 87) of the ASF entries have been borrowed directly from other databases, and the remainder have been calculated by applying conversion factors to borrowed values for food items in their raw form, to reflect changes in moisture content and nutrient losses due to cooking. Of all ASF entries, 88·9 % (*n* 112) are from the USA. In the West African FCT, directly-borrowed values constitute 41·6 % (*n* 47) and calculated values constitute 57·5 % (*n* 65) of all compositional data. Sources of data are given at the nutrient level, with a majority of ASF entries having been compiled from multiple sources. Approximately 31·1 % of data were derived from the USA, 24·5 % from European sources and 21·2 % from within the African continent. ASF entries in the Gambian database also include those composed of multiple data sources. Of the limited number of ASF reported (*n* 13), the vast majority are borrowed (84·6 %, *n* 11), with relatively balanced input from British databases, Gambian literature sources and Platt’s 1962 reference for foods ‘commonly used in tropical countries’^(^ ^31^ ^)^. Data sources for this last reference are not readily available, but are indicated to include a combination of primary analysis, published literature and alternative international databases. The Lesotho database identifies six of its total 283 entries to have been analysed directly, none of them ASF. This reference also has a strong reliance on data from alternative databases (88·2 %, *n* 45), with a predominance of ASF entries coming from South African (45·1 %, *n* 23) and US (35·3 %, *n* 18) references.

The Mozambique FCT is the only database examined to include ASF data from primary analysis of local foods (11·1 %, *n* 4). Dried forms of four species of fish (*pendhe*, *mirosse*, *sarabuanha* and *madambane*) were sourced from Mozambican markets in December 2008 and transported to Finland for analysis. A further four entries in the database have been calculated using these values.

The Tanzanian FCT does not report the sources of data for individual food items. The reference was developed using the World Food Dietary Assessment System, whereby information is imported from a series of six databases, considered representative of foods consumed in developing countries. The Kenyan database, which serves as the primary source for the Tanzanian FCT, uses nutrient values from references published in the 1960s^(^ ^25^ ^,^ ^31^ ^)^. Additional food items have been imported from databases from Egypt, India, Indonesia, Mexico and Senegal, as well as from US, UK and South African sources. There is no mention of data from in-country analysis.

### Comparison of nutrient content for poultry products

Compositional data from the UK^(^ ^28^ ^)^, USA^(^ ^29^ ^)^ and Australia^(^ ^30^ ^)^ were reviewed for selected poultry products. Differences were noted in the listing of food items in raw or cooked forms, the differentiation of various meat components of a carcass and, importantly, the inclusion of skin or fat. Table 3 details published values for proximates and selected micronutrients (vitamin A, vitamin B_12_, folate, vitamin E, Fe, Zn and Se) in raw forms of dark and light chicken meat, chicken liver and whole eggs.

**Table 3:** Published values from the UK, US and Australian food composition databases for selected nutrients in raw poultry products, per 100 g edible portion

		Food composition table		
	Units	UK	USA	Australia
*(a) Whole egg, chicken (raw)*		*Ref. 12-937*	*Ref. 01123*	*Ref. 03A10075*
Water	g	76·8	76·2	76·1
Energy	kJ	547	599	533
Protein	g	12·6	12·6	12·6
Lipids, total	g	9·0	9·5	8·5
Carbohydrates	g	Trace	0·7	0·3
Vitamin A, RAE	μg	126	160	130
Vitamin B_12_	μg	2·7	0·9	1·4
Folate, DFE	μg	47	47	110
Vitamin E	μg	1·3	1·1	2·2
Fe	mg	1·7	1·8	1·9
Zn	mg	1·1	1·3	1·1
Se	μg	23	31	26
*(b) Chicken liver (raw)*		*Ref. 18-411*	*Ref. 05027*	*Ref. 08D10194*
Water	g	76	76	75
Energy	kJ	386	496	466
Protein	g	17·7	16·9	16·9
Lipids, total	g	2·3	4·8	4·8
Carbohydrates	g	Trace	1	0
Vitamin A, RAE	μg	9700	3296	12007
Vitamin B_12_	μg	35·0	16·6	16·6
Folate, DFE	μg	995	588	1450
Vitamin E	μg	0·6	0·7	0·4
Fe	mg	9·2	9·0	9·8
Zn	mg	3·7	2·7	3·6
Se	μg		54·6	54·6
*(c) Chicken, light meat (raw)*		*Ref. 18-290*	*Ref. 05039*	*Ref. 08C10431*
Water	g	74	75	75
Energy	kJ	449	477	438
Protein	g	24	23	22
Lipids, total	g	1·1	1·7	1·6
Carbohydrates	g	0	0	0
Vitamin A, RAE	μg	Trace	27	8
Vitamin B_12_	μg	Trace	0·4	0·7
Folate, DFE	μg	14	4	0
Vitamin E	μg	0·1	0·2	2·2
Fe	mg	0·5	0·7	0·4
Zn	mg	0·7	1·0	0·7
Se	μg	12	18	25
*(d) Chicken, dark meat (raw)*		*Ref. 18-289*	*Ref. 05043*	*Ref. 08C10435*
Water	g	76	76	75
Energy	kJ	459	523	496
Protein	g	21	20	18
Lipids, total	g	3	4	5
Carbohydrates	g	0	0	0
Vitamin A, RAE	μg	20	22	19
Vitamin B_12_	μg	1·0	0·4	0·7
Folate, DFE	μg	9	10	14
Vitamin E	μg	0·2	0·2	0·6
Fe	mg	0·8	1·0	0·7
Zn	mg	1·7	2·0	1·5
Se	μg	14	14	20

Ref., reference code within database; RAE, retinol activity equivalents; DFE, dietary folate equivalents.

Beyond the expected variability between results of any two analyses, prominent and inconsistent differences were noted in a number of nutrients. Vitamin A levels in chicken liver varied widely between references, with retinol activity equivalents ranging from 3296 µg in the US database, to 9700 µg in the UK and to 12 007 µg in Australia. Variation was also seen in folate content of liver, with the UK and Australian references reporting levels 1·7 and 2·5 times higher than the US reference value, respectively. Vitamin B_12_ levels also varied across databases with the content in eggs reported to be 1·5 times higher in Australia and three times higher in the UK, compared with US values. In chicken liver, Australia- and US-reported levels of vitamin B_12_ were equivalent but less than half of the levels in the UK reference.

The implications of reported differences in nutrient content may be considered in terms of their contribution to an individual’s daily requirements. Reference nutrient intakes (RNI) for vitamin A, vitamin B_12_ and folate^(^ ^32^ ^)^ have been used to determine the contribution of a whole hard-boiled chicken egg to daily nutrient targets for people of various ages and physiological states, as presented in Table 4. Some figures are less relevant in a practical sense, but considerable variation can be seen in calculations using different data sources. US data indicate that a single egg would provide 20·5 % of the daily vitamin A requirements of a child aged 1–3 years, compared with 5·4 % using Australian data. For a pregnant woman, an egg represents 15·2 % of the RNI for folate using Australia-reported values, compared with 5·5 % according to the British database.

**Table 4:** Contribution of a 55-g, hard-boiled, whole chicken egg to reference nutrient intake (RNI, daily amount required to meet needs of 97·5 % of population) for vitamin A, vitamin B_12_ and folate

		% Contribution of one egg to RNI		
	RNI^(^ ^31^ ^)^ (µg/d)	UK (*Ref. 12-940*)	USA (*Ref. 01129*)	Australia (*Ref. 03A10062*)
*(a) Vitamin A*				
Child (1–3 years)	400	16·5	20·5	5·4
Female (15–50 years)	600	11·0	13·7	3·6
Pregnant female (15–50 years)	700	9·4	11·7	3·1
Breast-feeding female (15–50 years)	950	6·9	8·6	2·3
*(b) Vitamin B*_*12*_				
Child (1–3 years)	0·5	220·0	122·1	154·0
Female (15–50 years)	1·5	73·3	40·7	51·3
Pregnant female (15–50 years)	1·5	73·3	40·7	51·3
Breast-feeding female (15–50 years)	2·0	55·0	30·5	38·5
*(c) Folate*				
Child (1–3 years)	70	23·6	34·6	65·2
Female (15–50 years)	200	8·3	12·1	22·8
Pregnant female (15–50 years)	300	5·5	8·1	15·2
Breast-feeding female (15–50 years)	260	6·3	9·3	17·6

Ref., reference code within database.

Table 5 presents the required intake of fried chicken liver to meet RNI for these same three nutrients. Although not recommended for pregnant women in countries where vitamin A intakes commonly exceed RNI because of concerns about potential teratogenic effects, liver represents a rich dietary source of vitamin A in resource-poor settings. Consumption of a single fried chicken liver (approximately 42 g^(^ ^33^ ^)^) would meet the vitamin A requirements of a child aged 1–3 years for 13 d based on Australian reference values, compared with 4 d using US data. A breast-feeding mother’s daily folate needs are reported to be contained in 17 g of liver in Australia and 45 g in the USA.

**Table 5:** Contribution of fried chicken liver to reference nutrient intake (RNI, daily amount required to meet needs of 97·5 % of population) for vitamin A, vitamin B_12_ and folate

		Required intake (g) to meet RNI		
	RNI^(^ ^31^ ^)^ (µg/d)	UK (*Ref. 18-412*)	USA (*Ref. 05661*)	Australia (*Ref. 08D10161*)
*(a) Vitamin A*				
Child (1–3 years)	400	3·8	10·1	3·1
Female (15–50 years)	600	5·7	15·1	4·6
Pregnant female (15–50 years)	700	6·7	17·6	5·4
Breast-feeding female (15–50 years)	950	9·1	23·9	7·3
*(b) Vitamin B*_*12*_				
Child (1–3 years)	0·5	1·1	2·4	2·9
Female (15–50 years)	1·5	3·3	7·1	8·8
Pregnant female (15–50 years)	1·5	3·3	7·1	8·8
Breast-feeding female (15–50 years)	2·0	4·4	9·5	11·8
*(c) Folate*				
Child (1–3 years)	70	5·2	12·1	4·6
Female (15–50 years)	200	14·8	34·6	13·1
Pregnant female (15–50 years)	300	22·2	51·9	19·6
Breast-feeding female (15–50 years)	260	19·3	45·0	17·0

Ref., reference code within database.

## Discussion

Among the objectives of compiling national or regional FCT is the creation of reliable resources to meet the needs of users, which might include ‘government agencies, nutrition scientists, health and agriculture professionals, policymakers and planners, food producers, processors, retailers and consumers’^(^ ^34^ ^)^. In the international development arena, such tools are of particular value to those involved in programmes seeking food-based solutions to nutritional challenges. To assess the validity of existing references and establish their role in guiding efforts to improve food and nutrition security, this article considers (a) whether databases reflect the spectrum of foods consumed within a given country or region, (b) whether the origins of published data and details of the foods analysed make them appropriate for use in the intended area and (c) the reliability of original data sources.

Patterns in ASF consumption change over time, according to availability, access and a broad range of socio-economic and cultural factors. Short-term variation in diets may be seen between seasons, as well as longer-term variation according to climatic factors. Rigorous approaches to understanding diets, which consider the impact of seasons, agro-ecological zones, sex and socio-cultural factors, should underpin any national or regional food composition database. Introductory remarks to many of the FCT express an intention to present resources that represent local diets. The Gambian database reports an effort to cover foods typically consumed in rural areas, although the focus is on dishes (as prepared by the country’s predominant linguistic group, the Mandinka) rather than individual ingredients. The Ugandan resource, developed during a Consultative Group on International Agricultural Research project, includes all food items captured from two dietary intake surveys of women and children aged 6 months to 7 years, from three districts in the country’s Central and Eastern regions. Approaches to compiling food lists in other databases are less explicitly described. Although some similarities can be seen between national food supply data and the spectrum of food items in the FCT reviewed, it is unclear whether differences between databases reflect true differences in the types of foods consumed in various African countries.

There is a paucity of published data on the range of ASF consumed by African populations, including the breadth of carcass components eaten and the use of products from animals other than domestic livestock. Patterns of offal consumption vary with availability, cost, cultural beliefs, socio-economic attitudes and nutritional knowledge^(^ ^35^ ^)^. Although studies from Somalia suggest that many forms of offal are considered inferior meat because of their lower cost and are mainly consumed by women (with the exception of kidney and liver, which have an equivalent cost to muscle meat and are principally eaten by men), offal is reported to be widely consumed and considered both palatable and culturally acceptable^(^ ^35^ ^)^. With liver identified as among the best local food sources to improve dietary quality of young children^(^ ^36^ ^,^ ^37^ ^)^, there would be great advantage in broadening the range of micronutrient-rich organ meats in FCT to allow their inclusion in dietary recommendations in LMIC.

There has been some interest in the relative importance and nutrient content of edible indigenous plants in supplementing cereal-based diets of rural populations in Africa^(^ ^38^ ^,^ ^39^ ^)^; however, there remains much less reference in the published literature to the consumption of wild foods of animal origin. Although inter-country differences may exist in their availability and cultural acceptability, non-domesticated animals and insects contribute to nutrition security in many resource-poor settings. The harvest of wildlife provides a valuable source of meat for hundreds of millions of rural people living in poverty, often protecting against chronic under-nutrition where alternative sources of ASF are scarce or prohibitively expensive^(^ ^40^ ^,^ ^41^ ^)^. Insects have been an important wild source of protein and other macronutrients and micronutrients for thousands of years, particularly in remote rural areas and in tropical countries with high biodiversity^(^ ^42^ ^)^, and are the focus of current attention as an environmentally sustainable and nutritious complement to traditional protein sources^(^ ^43^ ^)^.

Projects seeking to document the food resources accessible by a given population have revealed great diversity in the range of items consumed throughout the year. An ethno-biological inventory of foods in Baringo District, Kenya, identified 226 edible species (thirty-eight ASF)^(^ ^44^ ^)^ and studies in Lusaka Province, Zambia, found 171 wild food species (eighty-seven ASF) that contribute to local diets^(^ ^45^ ^)^. Such findings have been generated from focus group discussions and interviews with community members to develop food lists in local languages, understand seasonal availability and food preparation methods, and working with national research institutions or museums to determine the scientific names and classifications of the food items identified^(^ ^44^ ^,^ ^45^ ^)^. Among the seven databases examined in this article, there is substantial variation in the inclusion of non-domesticated animals and insects. The Mozambican reference includes a single, wild ASF entry (rodent), whereas the West African reference provides data on caterpillars, locusts, winged ants and crickets. A lack of nutrient compositional data on the full spectrum of ASF from which a food-insecure household might benefit is a limitation of several references, as well as restricts the potential for knowledge of available food resources to be translated into guidelines for dietary improvement.

Despite the INFOODS programme’s strong focus on improving the quality, availability, reliability and use of food composition data globally, it remains evident that a majority of compositional data – including data published in recently released regional and national references – is drawn from other databases in geographically distant locations. Details of the source of data are sometimes lacking, and it is not always clear whether values are original data or borrowed or imputed from other sources. Of the FCT examined, only one included any original analysis of local ASF items and a large amount of data has been derived from outside Africa, and overwhelmingly from the USA. A lack of funding has consistently been highlighted by the INFOODS African Regional Data Centre (AFROFOODS) as the major obstacle for generating original analytical data^(^ ^46^ ^)^. The resulting reliance on existing references is evident in the 2010 Tanzanian FCT, based largely on databases compiled in the 1960s^(^ ^25^ ^,^ ^30^ ^)^ despite national capacity for nutrient analysis at institutions such as Sokoine University of Agriculture, the Tanzania Food and Nutrition Centre, Tanzania Veterinary Laboratory Agency and Tanzania Food and Drug Authority.

Even when original analytical and sampling methods are sound, this practice of ‘data recycling’ overlooks the potential for significant variation in the nutrient content of food items of both plant and animal origin. Introductory notes accompanying the UK database suggest the major source of variation in meat composition to be differing fat content, both as a result of husbandry and food preparation techniques^(^ ^27^ ^)^. Most nutrients are said to be affected, due to differences in their distribution in lean meat and associated fat. Elsewhere, genetic strain has been identified as a crucial factor affecting meat quality^(^ ^47^ ^,^ ^48^ ^)^. Such observations affirm the limitations of applying international reference data to indigenous breed livestock raised in low-input systems in resource-poor settings.

Taking chickens as an example, marked physical differences are evident between local breed poultry raised in small free-ranging flocks in rural African communities, typically largely or wholly reliant on environmental feed sources, and those of genetic lines selected for rapid growth and high feed conversion ratios, reared on balanced feed rations in commercial production systems. Among commercial poultry producers, it is recognised that nutrient requirements differ significantly between broiler (meat-producing) and layer (egg-producing) birds and between different stages of the life cycle^(^ ^49^ ^)^. Scavenging village chickens may achieve a balanced diet under some conditions, eating crop residues after the harvest and fresh plant material, insects, worms and molluscs during the wet season. In areas with unimodal, irregular or limited rainfall, however, there are likely to be times of environmental food shortage during the dry season, when birds are at risk of inadequate nutrition^(^ ^50^ ^)^.

Documented changes in the carcass composition and yield of commercial breed chickens over time have been attributed to genetic selection^(^ ^51^ ^)^, confinement of domestic livestock and provision of high-energy feed^(^ ^52^ ^)^. Although poultry has historically been regarded as a lean option compared with red meat^(^ ^53^ ^)^, the modern broiler chicken has been reported to provide several times more energy from fat than from protein and contains a ratio of *n*-6:*n*-3 fatty acids of as high as 9:1 – a shift away from the lower ratio advised for decreasing the risk of CHD^(^ ^54^ ^)^.

Interest in the impact of livestock production systems on the nutrient content of food products has grown in recent years, as consumer concerns about welfare standards have prompted a shift away from traditional battery cages for hens and indoor housing of pigs towards free-range systems. The effect on nutrient levels remains unresolved, with some studies showing no significant differences^(^ ^55^ ^,^ ^56^ ^)^ and others attributing variation to diet^(^ ^57^ ^)^. In the case of eggs, such studies have focused on cholesterol and PUFA levels, and not on other micronutrients of public health significance. As for any food, variation in water content is a major determinant of the nutrient density of eggs and relates to both the ratio of yolk:white and the solids content of each component^(^ ^16^ ^,^ ^58^ ^)^. Evaluation of the effect of egg size, hen age and genetic strain on solid content has shown smaller eggs and older hens to be associated with a higher solid content^(^ ^58^ ^)^. This might appear to be a favourable finding for the nutrient density of village chickens’ eggs, typically smaller and produced by older hens than their commercial counterparts; however, the absence of published data on the nutrient content of poultry products from resource-poor settings remains a major gap.

Also of concern is the variation that exists between compositional data from developed nations, including in micronutrients of public health significance. As databases continue to be revised and updated, individual entries are often comprised of compositional data from multiple analyses conducted years or decades apart. Data on the vitamin A content of raw chicken liver are based on analyses from the early 1980s, 2003 and 2005 for the UK, US and Australian references, respectively. Details of analytical techniques are found in general comments accompanying each database, and suggest no methodological reason for a 3·5-fold difference between US and Australian values. The apparent disparity between nutrient levels in the products of commercially reared chickens from different nations – of similar genetic lines, each receiving feed intended to meet nutritional requirements while minimising excessive nutrient excretion – further highlights the variability that might be expected in the nutrient content of scavenging chickens in resource-poor settings.

Calculations to determine the required daily intake of ASF items to meet specific nutrient requirements may appear to undermine the significance of data variability – for example, when considering the difference between a 7 and 24-g portion of chicken liver to meet a breast-feeding mother’s daily vitamin A requirements (using Australian and US references, respectively; Table 4). The equivalent vitamin A intake from plant-based sources would equate to over 700 g of fresh papaya^(^ ^27^ ^)^, almost 150 g of cooked spinach^(^ ^27^ ^)^ or approximately 220 g of boiled orange-fleshed sweet potato^(^ ^20^ ^)^. In this context, where intakes of vitamin A are marginal or low and even small portions of ASF have the potential to meet an individual’s requirements, 2–3-fold differences in compositional data become more relevant. The value of reliable data on locally available resources should not be underestimated.

### Conclusion

Access to current, relevant and reliable data on the nutrient content of food items is fundamental for those working to implement sustainable responses to global food and nutrition security challenges. In the African region, several databases released in recent years provide readily accessible sources of compositional data. There are significant limitations, however, when these references do not reflect the full range of food items that might be consumed by members of a food-insecure household, including invertebrates, non-domestic animals, offal and other carcass components, as well as indigenous and non-cultivated plants. New references rarely provide new data, and the volume of information ‘recycled’ from other databases, which are often from analyses conducted decades previously and sourced from high-income countries, limits their usefulness. In the case of ASF, it is important to acknowledge the variation in the nutrient content likely to be associated with differences in livestock breeds, management systems, diet, seasons and environments. Less recognised is the variation that exists between the nutrient content of equivalent ASF items reported in databases of high-income nations, and the implications for borrowing data from one reference over another.

It is clear that limitations of FCT are not restricted to resource-poor settings. There are global efforts to standardise and harmonise food composition data, including work coordinated by the European Food Information Resource association and the FAO to establish protocols and improve data quality and database searchability^(^ ^59^ ^)^. Together with strong partnerships between research institutions, industry bodies and disciplinary areas, these efforts may enable existing data from LMIC (e.g. generated by universities or animal feed companies) to be assessed for quality and integrated into national or regional databases. Research and development projects that use food-based approaches might also be encouraged to undertake analysis of local items of interest, with a view of incremental expansion of national databases.

The INFOODS programme and its regional data centres have done much to raise the profile of FCT activities and support capacity-building through training courses and institutional collaborations; however, there remains a strong need for funding – both through the support of donor agencies and commitment from national governments – to generate new location-specific compositional data. Accurate information on the nutrient content of locally available food items will better guide work in nutrition-sensitive and cost-efficient interventions and enable the development of meaningful guidelines for improving dietary adequacy.
